# Pioglitazone Ameliorates Smooth Muscle Cell Proliferation in Cuff-Induced Neointimal Formation by Both Adiponectin-Dependent and -Independent Pathways

**DOI:** 10.1038/srep34707

**Published:** 2016-10-05

**Authors:** Tetsuya Kubota, Naoto Kubota, Hiroyuki Sato, Mariko Inoue, Hiroki Kumagai, Tomokatsu Iwamura, Iseki Takamoto, Tsuneo Kobayashi, Masao Moroi, Yasuo Terauchi, Kazuyuki Tobe, Kohjiro Ueki, Takashi Kadowaki

**Affiliations:** 1 Department of Diabetes and Metabolic Diseases, Graduate School of Medicine, University of Tokyo, Tokyo 113-8655, Japan; 2 Laboratory for Metabolic Homeostasis, RIKEN Center for Integrative Medical Sciences, Kanagawa, 230-0045, Japan; 3 Department of Clinical Nutrition, National Institute of Health and Nutrition, Tokyo 162-8636, Japan; 4 Division of Cardiovascular Medicine, Toho University Ohashi Medical Center, Tokyo 153-8515, Japan; 5 Department of Clinical Nutrition Therapy, University of Tokyo, Tokyo 113-8655, Japan; 6 Department of Physiology and Morphology, Institute of Medicinal Chemistry, Hoshi University, Tokyo 142-8501, Japan; 7 Department of Diabetes and Endocrinology, Yokohama City University, School of Medicine, Kanagawa 236-0004, Japan; 8 First Department of Internal Medicine, Faculty of Medicine, University of Toyama, Toyama, 930-0194, Japan

## Abstract

The aim of this study is to elucidate to what degree adiponectin is involved in TZD-mediated amelioration of neointimal formation. We investigated the effect of 3- or 8-weeks’ pioglitazone on cuff-induced neointimal formation in adiponectin-deficient (APN-KO) and wild-type (WT) mice. Pioglitazone for 3 weeks reduced neointimal formation in the WT mice with upregulation of the plasma adiponectin levels, but failed to reduce neointimal formation in the APN-KO mice, suggesting that pioglitazone suppressed neointimal formation by adiponectin-dependent mechanisms. Pioglitazone for 3 weeks suppressed vascular smooth muscle cell (VSMC) proliferation and increased AdipoR2 expression in the WT mice. *In vitro,* globular adiponectin activated AMPK through both AdipoR1 and AdipoR2, resulting in the inhibition of VSMC proliferation. Interestingly, 8-weeks’ pioglitazone was reduced neointimal formation in APN-KO mice to degree similar to that seen in the WT mice, suggesting that pioglitazone can also suppress neointimal formation via a mechanism independent of adiponectin. Pioglitazone for 8 weeks completely abrogated the increased VSMC proliferation, along with a reduction of cyclin B1 and cyclin D1 expressions and cardiovascular risk profile in the APN-KO mice. *In vitro,* pioglitazone suppressed these expressions, leading to inhibition of VSMC proliferation. Pioglitazone suppresses neointimal formation via both adiponectin-dependent and adiponectin-independent mechanisms.

Thiazolidinediones (TZDs) have been shown to act as insulin sensitizers in both animals and humans with obesity-linked insulin resistance and type 2 diabetes^1^,^2^,^3^,^4^, and are used as one class of therapeutic agents in the treatment of type 2 diabetes mellitus. In addition to controlling glycemia, TZDs have also been shown to have beneficial effects on other cardiovascular risk factors, such as dyslipidemia and hypertension^5^. Moreover, TZDs have been demonstrated to suppress atherosclerosis independent of their effects on these risk factors^6^,^7^,^8^,^9^,^10^, suggesting that TZDs may have direct actions on the vasculature. A previous meta-analysis indicated that TZDs reduced the risk of repeat revascularization in patients undergoing percutaneous coronary intervention (PCI)^11^. Pioglitazone, one of the representative TZDs, was shown in a PRO active study to reduce the composite of all-cause mortality, non-fatal myocardial infarction and stroke in patients with type 2 diabetes who are at a high risk of developing macrovascular events^12^.

TZDs activate peroxisome proliferator-activated receptor gamma (PPARγ), which is a member of the nuclear hormone receptor superfamily, and is expressed on major cells involved in the development of atherosclerosis, including endothelial cells, vascular smooth muscle cells (VSMCs) and monocytes/macrophages^13^,^14^,^15^. Pioglitazone failed to attenuate the massive vascular lesion formation in the transplanted carotid artery from VSMC-specific PPARγ-deficient mice^16^. Pioglitazone also failed to suppress atherosclerosis in SMC-specific PPARγ/low-density lipoprotein receptor (LDLR) double-deficient mice^17^. A recent study revealed that pioglitazone modulated VSMC proliferation via PPARγ^18^. These data suggest that pioglitazone suppresses atherosclerosis through the PPARγ expressed in VSMCs.

In addition to the direct vascular effects of TZDs, TZDs have been shown to upregulate adiponectin expression in white adipose tissue and to increase the plasma adiponectin levels, which is well known to have anti-atherogenic properties^19^. We and others have previously demonstrated that adiponectin-deficient (APN-KO) mice show neointimal formation in response to cuff- or wire-injury, and that adenovirus vector-mediated adiponectin transfection suppressed the VSMC proliferation and migration^20^,^21^,^22^. Many epidemiological studies have revealed a relationship between plasma adiponectin levels and the risk of cardiovascular events^23^,^24^. Moreover, two distinct adiponectin receptors have been identified: AdipoR1 and AdipoR2^25^. Although AdipoR1 and AdipoR2 expression levels show tissue-specific differences, both are expressed in the vasculature, including the VSMCs and endothelial cells. The expression levels of AdipoR1 and AdipoR2 have been shown to be significantly reduced in the coronary arteries of animal models of type 2 diabetes^26^. These findings suggest that the anti-atherogenic actions of TZDs are adiponectin-dependent. However, whether the TZD-induced increase in plasma adiponectin is causally involved in the anti-atherogenic effects of TZDs has not yet been addressed.

In the present study, we used APN-KO mice to investigate whether pioglitazone, one of the representative TZDs, is capable of ameliorating neointimal formation in the absence of adiponectin. In the wild-type (WT) mice, pioglitazone treatment was associated with a significant increase of the plasma adiponectin from 1 week onward, and 3-weeks’ treatment was associated with attenuation of cuff-induced neointimal formation. On the other hand, in the APN-KO mice, the cuff-induced neointimal formation remained unchanged after 3 weeks of pioglitazone treatment, suggesting that the pioglitazone-induced suppression of cuff-induced neointimal formation in the WT mice was dependent on adiponectin. Cuff-induced neointimal formation is associated with the proliferation of α-smooth muscle actin-positive cells, and the VSMC proliferation was significantly suppressed after 3-weeks’ pioglitazone treatment in the WT mice, but not APN-KO mice. Moreover, increased AdipoR2 expression levels were observed in the neointima after 3-weeks’ pioglitazone treatment, suggesting that this upregulation is augmented adiponectin’s action. Adiponectin attenuated the VSMC proliferation induced by platelet-derived growth factor (PDGF)-BB through the AdipoR1- and AdipoR2- AMPK pathways in human aortic SMCs (HASMCs). Interestingly, however, both WT and APN-KO mice exhibited similar significant improvement of neointimal formation after 8-weeks’ pioglitazone treatment, associated with a reduction of the cardiovascular risk profile. These data suggest that pioglitazone can also suppress neointimal formation independently of its actions mediated by adiponectin. The increased VSMC proliferation in the APN-KO mice was completely reversed after 8 weeks of pioglitazone treatment, along with a reduction of cyclinB1 and cyclinD1 expression levels. Pioglitazone directly inhibited VSMC proliferation via suppression of these expression levels in HASMCs. Taken together, pioglitazone-induced amelioration of neointimal formation may occur via both adiponectin-dependent and adiponectin-independent mechanisms.

## Results

### Pioglitazone treatment for 3 weeks suppressed neointimal formation induced by cuff injury in an adiponectin-dependent manner, accompanied by augmentation of AdipoR2 expression

**Figure 1:**
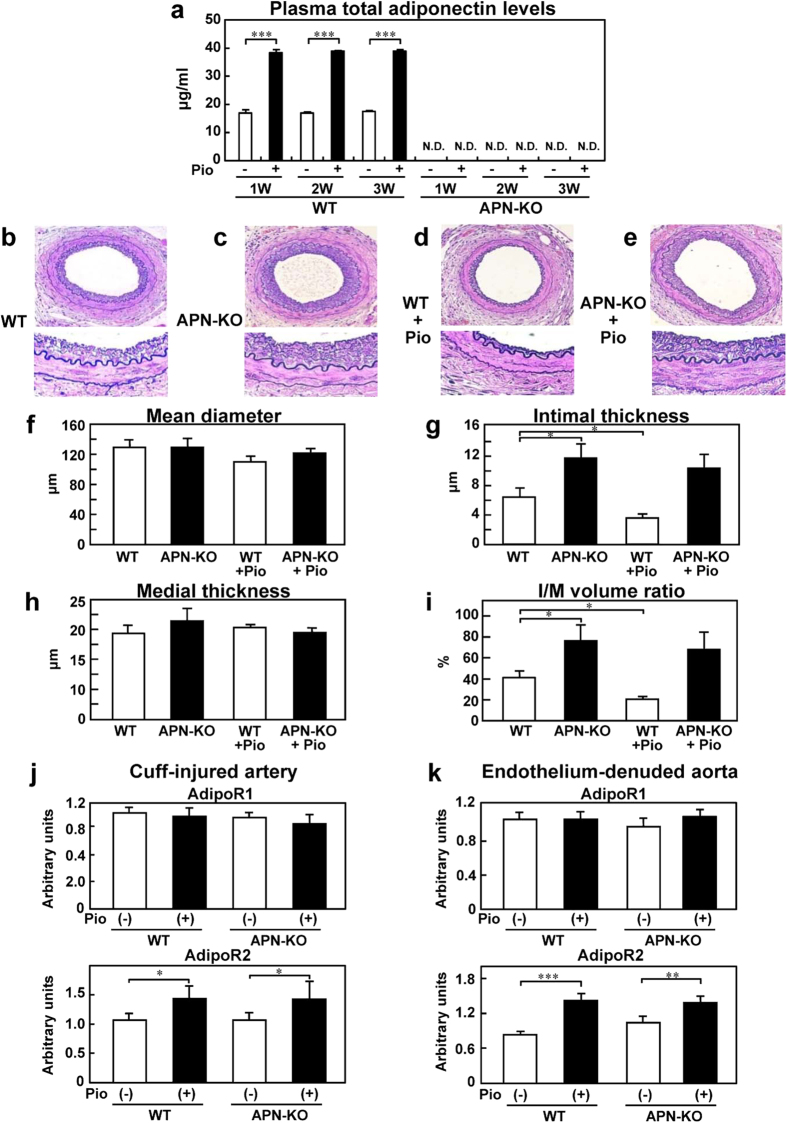
Pioglitazone treatment for 3 weeks inhibited cuff-induced neointimal formation in the WT, but not APN-KO mice. (**a**) Plasma total adiponectin levels after 3 weeks of treatment with pioglitazone in the WT and APN-KO mice (n = 5). Representative cuff-injured vessels from WT (**b**) APN-KO (**c**) WT+pioglitazone (Pio) (**d**) and APN-KO+Pio (**e**) mice after 3-weeks’ pioglitazone treatment (upper; ×40, lower; ×400). Morphometric analysis of the blood vessels in the WT and APN-KO mice; Mean diameter (**f**), Intimal thickness (**g**) Medial thickness (**h**) Intima-to-media (I/M) volume ratio (**i**) in response to vessel injury after 3-weeks’ treatment with pioglitazone (n = 8–12). Expression levels of AdipoR1 and AdipoR2 in the cuff-injured artery (**j**) and endothelium-denuded aorta (**k**) in the WT and APN-KO mice (n = 5–8). N.D. is “not-detected”. Data are mean ± SEM. *P < 0.05; ***P < 0.001.

Significant increase of the plasma total adiponectin levels was observed from 1 week onward after the start of pioglitazone treatment in the WT mice, but not APN-KO mice (Fig. 1a). Furthermore, increased plasma high-molecular-weight (HMW) adiponectin levels were also observed from 1 week after the start of pioglitazone treatment in the WT mice, but not in the APN-KO mice (Supplementary Fig. S1). To investigate whether this increase of the plasma adiponectin was associated with suppression of neointimal formation, cuff injury was induced in the femoral artery of the WT and APN-KO mice during 3 weeks the course of pioglitazone treatment. The APN-KO mice showed a significant cuff-induced increase of the intimal thickness and intimal/medial volume ratio (Fig. 1b,c,g,i), as previously reported^20^. Pioglitazone treatment for 3 weeks significantly reduced the intimal thickness and intimal/medial volume ratio in the WT mice, but not in the APN-KO mice (Fig. 1d,e,g,i). No significant differences were observed in the mean diameter or medial thickness among the WT and APN-KO mice either before and after pioglitazone treatment (Fig. 1f,h). Since PPARγ agonists have been reported to upregulate AdipoR2 mRNA expression, we next investigated the expression levels of AdipoR1 and AdipoR2 mRNA in the cuff-induced neointimal formation after 3 weeks of pioglitazone treatment. Although the AdipoR1 mRNA expression levels did not differ among the four groups, pioglitazone treatment for 3 weeks was associated with increased AdipoR2 mRNA expression levels in the cuff-induced neointimal formation in both the WT and APN-KO mice (Fig. 1j). Increase of the AdipoR2 mRNA expression level was also observed in the endothelium-denuded aorta of both WT and APN-KO mice after 3 weeks of pioglitazone treatment (Fig. 1k). These data suggest that 3 weeks of pioglitazone treatment suppressed neointimal formation in an adiponectin-dependent manner, accompanied by augmentation of AdipoR2 expression.

### Pioglitazone treatment inhibited VSMC proliferation in the cuff-induced neointimal formation in an adiponectin-dependent manner

**Figure 2:**
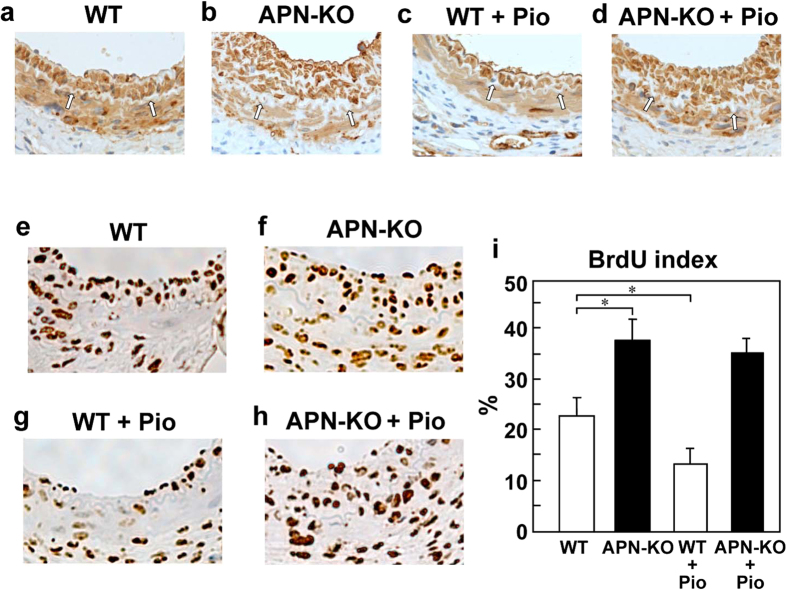
Pioglitazone treatment for 3 weeks suppressed cuff-induced VSMC proliferation in the WT mice, but not APN-KO mice. Representative immunohistochemical staining for alpha smooth muscle actin of injured vessels obtained from WT (**a**) APN-KO (**b**), WT+Pio (**c**) and APN-KO+Pio (**d**) mice after 3-weeks’ treatment with pioglitazone. Representative BrdUrd staining for SMC proliferation (**e–h**) and ratio of the number of BrdUrd positive/BrdUrd- negative cells (**i**) in the arterial specimens obtained from the WT, APN-KO, WT+Pio and APN-KO+Pio mice after 3-weeks’ treatment with pioglitazone (n = 5–6). Arrows show the internal elastic lamina. Data are mean ± SEM. *P < 0.05.

The neointimal thickness was next assessed by α-smooth muscle actin staining. The majority of cells in the neointima of the cuff-injured vessels, as well as in the media, showed positive staining for α-smooth muscle actin (Fig. 2a–d), as previously reported^27^. These findings indicate that the cuff-induced neointimal formation is composed predominantly of VSMCs. We performed immunohistological analyses to assess the proliferation of VSMCs in the cuff-induced neointimal formation. The percentage of cells incorporating BrdU was significantly higher in the APN-KO mice than in the WT mice (Fig. 2e,f,i). Pioglitazone treatment for 3 weeks significantly reduced BrdU incorporation in the cuff-induced neointimal formation in the WT mice, but not in the APN-KO mice (Fig. 2g–i). These data suggest that the proliferation of the VSMCs in response to cuff injury was suppressed by pioglitazone treatment for 3 weeks through an adiponectin-dependent pathway.

### Adiponectin suppressed PDGF-BB-induced VSMC proliferation via AdipoR1- and AdipoR2-mediated AMPK activation

**Figure 3:**
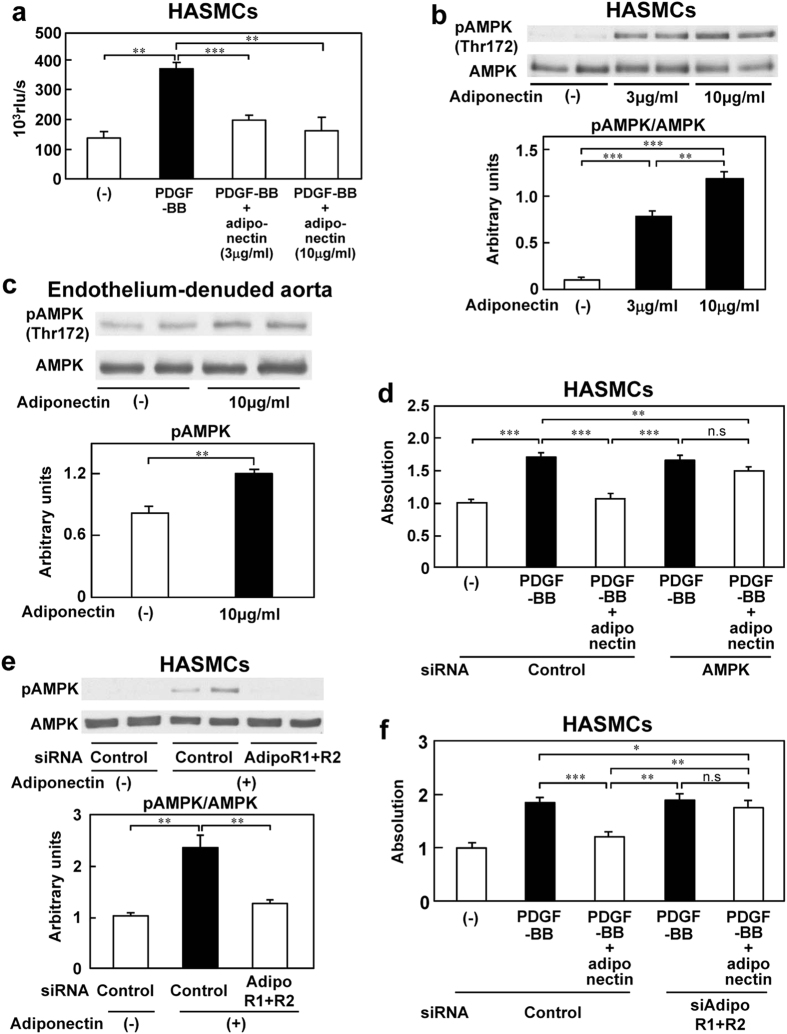
Adiponectin suppressed PDGF-BB induced HASMC proliferation through both AdipoR1- and AdipoR2-mediated AMPK activation. (**a**) Effect of globular adiponectin on PDGF-BB-induced BrdU incorporation in the HASMCs (n = 4–8). Phosphorylation of AMPK in the presence or absence of globular adiponectin in the HASMCs (**b**) (n = 4) and endothelium-denuded aorta in the WT mice (**c**) (n = 4). (**d**) Downregulation of AMPK by siAMPK abrogated the antiproliferative effect of globular adiponectin (n = 10). (**e**) Downregulation of both AdipoR1 and AdipoR2 completely abolished AMPK phosphorylation in the HASMCs (n = 4). (**f**) Downregulation of both AdipoR1 and AdipoR2 abrogated the antiproliferative effect of globular adiponectin in the HASMCs (n = 10). Data are mean ± SEM. *P < 0.05; **P < 0.01; ***P < 0.001.

To elucidate the adiponectin-dependent mechanisms underlying the suppression of VSMC proliferation by pioglitazone, we conducted an experiment using HASMCs. In previous studies in a rat carotid model, increase of PDGF-BB expression was demonstrated in the neointima formed after balloon injury, and a selective antagonist of PDGF-BB significantly reduced the neointimal formation^28^,^29^,^30^. Thus, we observed adiponectin actions on PDGF-BB-induced VSMC proliferation in the HASMCs. In the BrdU incorporation assay, increased DNA synthesis induced by PDGF-BB was significantly suppressed by globular adiponectin, suggesting that adiponectin inhibits VSMC proliferation (Fig. 3a). Consistent with these data, the cell counting kit (CCK)-8 assay and WST-1 cell proliferation assay also revealed significant suppression by globular adiponectin of the enhanced cell viability induced by PDGF-BB (Supplementary Fig. S2a,b). Since adiponectin can activate AMPK in the liver and skeletal muscle^25^, we investigated the phosphorylation of AMPK after stimulation with globular adiponectin in HASMCs or endothelium-denuded mouse aorta. Stimulation with globular adiponectin increased AMPK phosphorylation in both the HASMCs and endothelium-denuded mouse aorta (Fig. 3b,c). When the AMPK protein levels were reduced by transfection of siAMPK into the HASMCs, the PDGF-BB-stimulated VSMC proliferation was no longer suppressed (Fig. 3d and Supplementary Fig. S2c). These results suggest that AMPK activation by adiponectin suppressed PDGF-BB-induced VSMC proliferation in the HASMCs.

Since adiponectin is reported to activate AMPK via the adiponectin receptors, AdipoR1 and AdipoR2^31^, we investigated whether adiponectin also activated AMPK via AdipoR1 and AdipoR2 in the HASMCs. The expression of AdipoR1 mRNA was downregulated following transfection of AdiopR1 siRNA, whereas the AdipoR2 mRNA levels remained unchanged (Supplementary Fig. S2d). Similarly, siAdipoR2 was used to reduce the expression levels of AdipoR2 mRNA without affecting the AdipoR1 mRNA expression levels (Supplementary Fig. S2d). Adiponectin-stimulated AMPK phosphorylation was partially disturbed by knockdown of AdipoR1 or AdipoR2 in the HASMCs (Supplementary Fig. S2e). Knockdown of both AdipoR1 and AdipoR2 almost completely suppressed the phosphorylation of AMPK by adiponectin (Fig. 3e and Supplementary Fig. S2f). Moreover, the effect of adiponectin of inhibiting PDGF-BB-stimulated VSMC proliferation was completely abrogated in the HASMCs (Fig. 3f). These data suggest that adiponectin suppresses PDGF-BB-stimulated VSMC proliferation through AdipoR1- and AdipoR2-mediated AMPK activation.

### Pioglitazone treatment improved the cardiovascular risk profile in an adiponectin-independent manner

**Figure 4:**
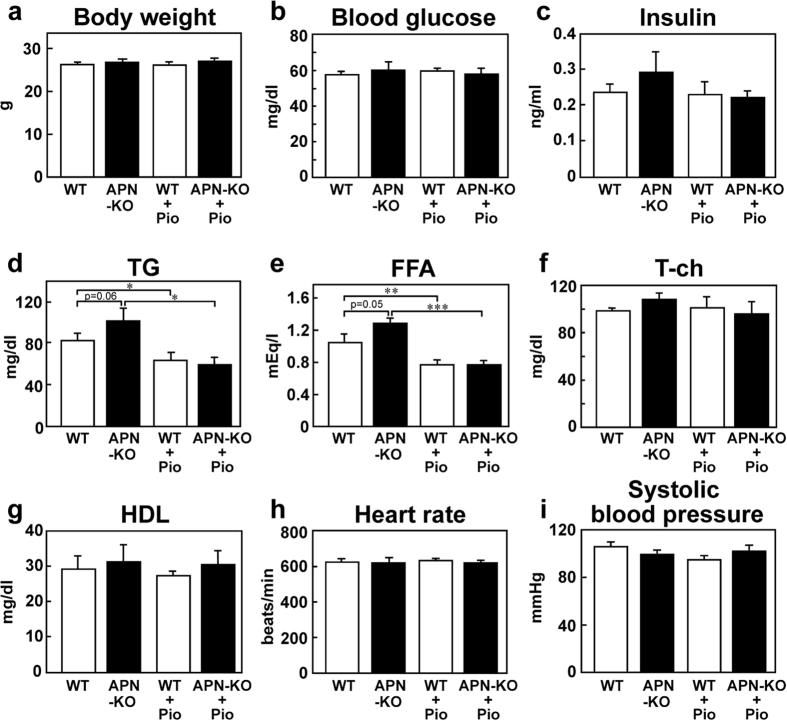
Pioglitazone treatment for 3 weeks improved the cardiovascular risk profile via an adiponectin-independent pathway (**a**) Body weight in the WT and APN-KO mice (n = 6–8). (**b**) Fasting blood glucose levels in the WT and APN-KO mice (n = 6–8). (**c**) Fasting plasma insulin levels in the WT and APN-KO mice (n = 6–8). (**d**) Fasting plasma triglyceride (TG) levels in the WT and APN-KO mice (n = 6–8). (**e**) Fasting plasma free fatty acid (FFA) levels in the WT and APN-KO mice (n = 6–8). (**f**) Fasting plasma total cholesterol (T-ch) levels in the WT and APN-KO mice (n = 6–8). (**g**) Fasting plasma high-density lipoprotein (HDL) levels in the WT and APN-KO mice (n = 6–8). (**h**) Heart rate in the WT and APN-KO mice (n = 5–9). (**i**) Systolic blood pressure in the WT and APN-KO mice (n = 5–9). Data are mean ± SEM. *P < 0.05; **P < 0.01; ***P < 0.001.

The body weight, blood glucose, plasma insulin level and plasma lipid profile were measured after 3 weeks of pioglitazone treatment. No significant differences were observed in the body weight, fasting blood glucose or plasma insulin levels among the four groups (Fig. 4a–c). Plasma triglyceride (TG) and free fatty acid (FFA) levels tended to be higher in the APN-KO mice than in the WT mice (Fig. 4d,e), as previously reported^20^. Plasma TG and FFA levels in the APN-KO mice decreased to a degree similar to that obtained after 3 weeks of pioglitazone treatment in the WT mice (Fig. 4d,e), suggesting that these reductions occurred in an adiponectin-independent manner. No significant differences were observed in the total cholesterol (T-ch) or high-density lipoprotein (HDL) levels among the four groups (Fig. 4f,g). The heart rate and systolic blood pressure also did not differ among four groups after 3 weeks of pioglitazone treatment (Fig. 4h,i).

### Pioglitazone treatment for 8 weeks significantly decreased cuff-induced neointimal formation in an adiponectin-independent manner

**Figure 5:**
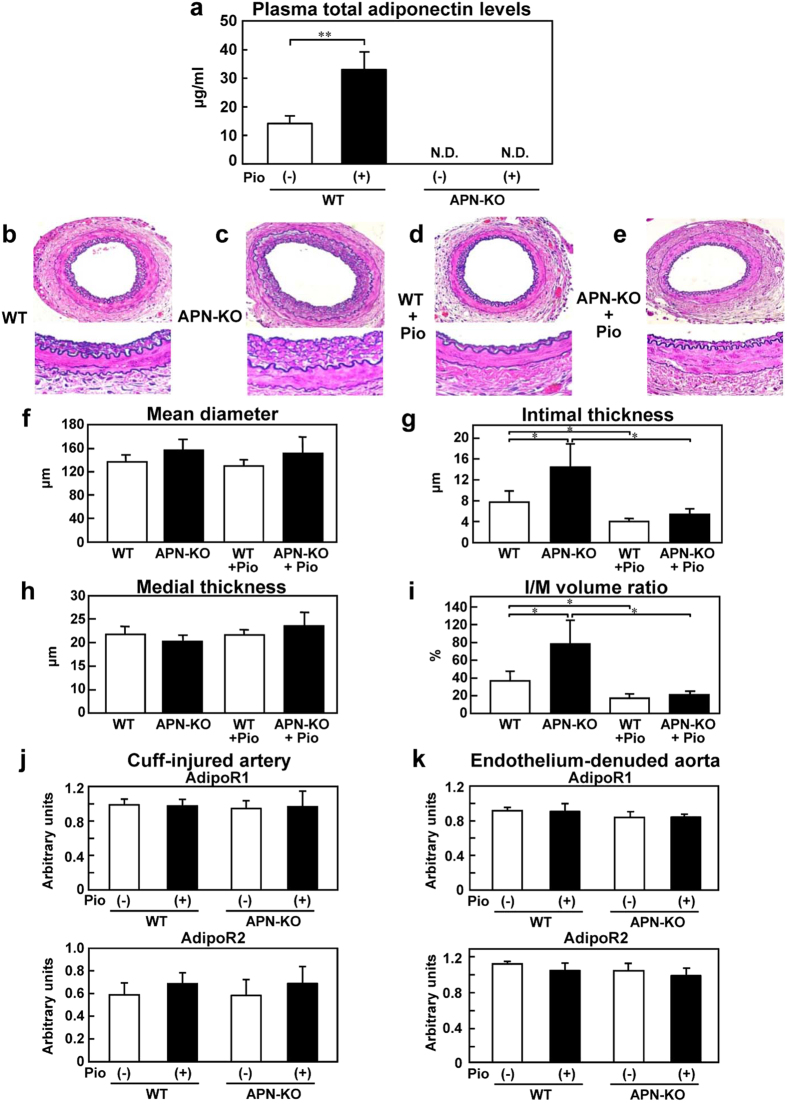
Pioglitazone treatment for 8 weeks inhibited cuff-induced neointimal formation in both WT and APN-KO mice. (**a**) Plasma total adiponectin levels after 8-weeks’ treatment with pioglitazone in the WT and APN-KO mice (n = 5). Representative cuff-injured vessels from WT (**b**) APN-KO (**c**) WT+pioglitazone (Pio) (**d**) and APN-KO+Pio (**e**) mice after 8-weeks’ treatment with pioglitazone (upper; ×40, lower; ×400). Morphometric analysis of the injured blood vessels; Mean diameter (**f**) Intimal thickness (**g**) Medial thickness (**h**) Intima-to-media (I/M) volume ratio (**i**) in response to 8-weeks’ treatment with pioglitazone in the WT and APN-KO mice (n = 9–12). The expression levels of AdipoR1 and AdipoR2 in the cuff-injured artery (**j**) and endothelium-denuded aorta (**k**) in the WT and APN-KO mice (n = 4–8). N.D. is “not-detected”. Data are mean ± SEM. *P < 0.05; **P < 0.01.

To investigate the effect of long-term pioglitazone treatment on cuff-induced neointimal formation, we administered pioglitazone to APN-KO and WT mice for 8 weeks. While the plasma total adiponectin levels significantly increased in the WT mice after 8 weeks of pioglitazone treatment to a similar degree as that at 3 weeks, no such increase was detectable in the APN-KO mice (Fig. 5a). Moreover, a similar degree of increase of the plasma HMW adiponectin levels was found after 8 weeks of pioglitazone treatment to that after 3 weeks of treatment in the WT mice, but not in the APN-KO mice (Supplementary Fig. S3). Cuff-induced increases of the intimal thickness and intimal/medial volume ratio were significantly higher in the APN-KO mice than in the WT mice (Fig. 5b,c,g,i). Interestingly, however, pioglitazone treatment for 8 weeks significantly reduced the cuff-induced increase of the intimal thickness and intimal/medial volume ratio in the APN-KO mice to a degree similar to that seen in the WT mice treated with pioglitazone for 8 weeks (Fig. 5d,e,g,i). There were no significant differences in the mean vessel diameter or medial thickness among the four groups (Fig. 5f,h). In contrast to the case after 3 weeks of pioglitazone treatment, no significant differences in either AdiopR1 expression or AdipoR2 expression in the cuffed artery or endothelium-denuded mouse aorta were observed among the four groups after 8 weeks of pioglitazone treatment (Fig. 5j,k). These data suggest that 8 weeks of pioglitazone treatment caused a significant decrease of cuff-induced neointimal formation via an adiponectin-independent mechanism.

### Pioglitazone suppressed VSMC proliferation via regulation of cyclin B1 and cyclin D1 expression levels

**Figure 6:**
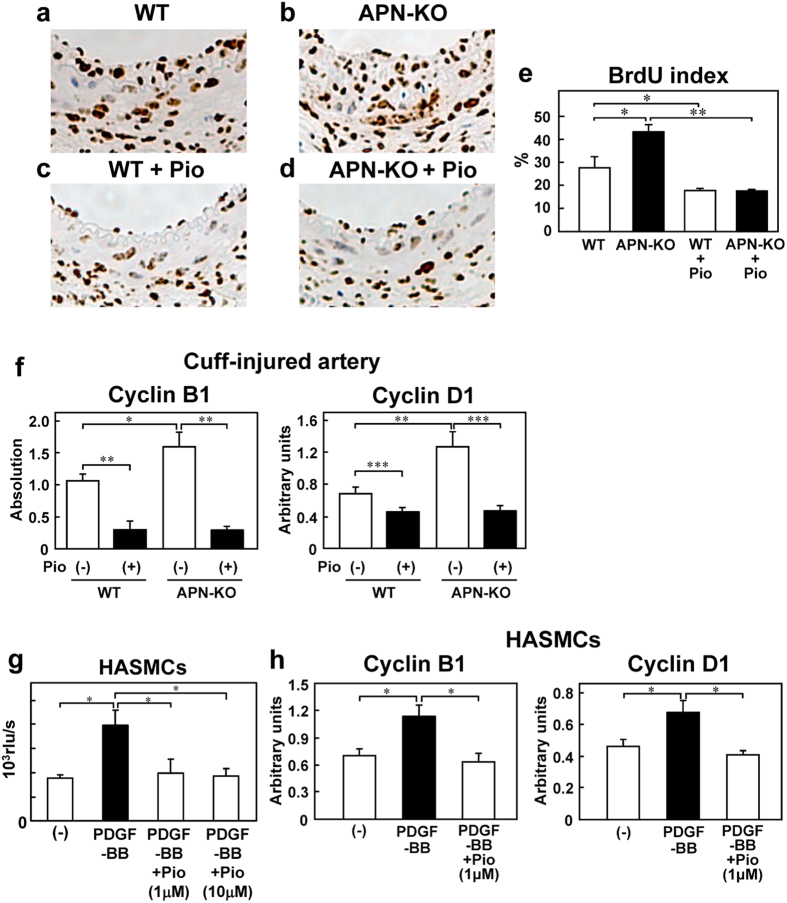
Pioglitazone treatment for 8 weeks suppressed cuff-induced VSMC proliferation in both WT and APN-KO mice. Representative BrdUrd staining for SMC proliferation (**a–d**) and ratio of the number of BrdUrd-positive/BrdUrd-negative cells (**e**) in the cuff-injured vessels obtained from WT, APN-KO, WT+Pio and APN-KO+Pio mice after 8-weeks’ treatment with pioglitazone (n = 5–6). (**f**) Expression levels of cyclin B1 and cyclin D1 in the cuff-injured artery in the WT and APN-KO mice (n = 6–7). (**g**) Effect of pioglitazone on PDGF-BB-induced BrdU incorporation in the HASMCs (n = 4–8). (**h**) Pioglitazone reduced the expression levels of cyclin B1 and cyclin D1 after PDGF-BB stimulation in the HASMCs (n = 4). Data are mean ± SEM. *P < 0.05; **P < 0.01; ***P < 0.001.

Following 8-weeks’ treatment with pioglitazone, similar to the observation after 3-weeks’ treatment, the majority of the cells in the neointima of the cuff-injured vessels showed positive staining for α-smooth muscle actin (Supplementary Fig. S4a–d). Although the percentage of cells incorporating BrdU in the APN-KO mice was significantly higher than that in the WT mice, the percentage decreased significantly following 8 weeks of pioglitazone treatment to the same level as that in the pioglitazone-treated WT mice (Fig. 6a–e). These findings suggest that pioglitazone treatment is also capable of suppressing VSMC proliferation by an adiponectin-independent mechanism. Moreover, we investigated the expression levels of cyclin B1 and cyclin D1 in the cuff-injured artery in both the WT and APN-KO mice after 8 weeks of pioglitazone treatment. The expression levels of cyclin B1 and cyclin D1 in the cuffed artery were higher in the APN-KO mice than in the WT mice without pioglitazone treatment (Fig. 6f). After pioglitazone treatment for 8 weeks, these expression levels in the cuff-injured artery of the APN-KO mice decreased to levels similar to those seen in the WT mice treated with pioglitazone for 8 weeks (Fig. 6f). We investigated whether pioglitazone directly mediated VSMC proliferation by regulating the expression levels of cyclin B1 and cyclin D1. In the BrdU incorporation assay, increased DNA synthesis in the HASMCs induced by PDGF-BB was completely abolished by treatment with pioglitazone, suggesting that pioglitazone inhibits the proliferation of the VSMCs (Fig. 6g). Consistent with these data, the CCK-8 assay and WST-1 cell proliferation assay also revealed significant suppression by pioglitazone of the enhanced cell viability induced by PDGF-BB (Supplementary Fig. S4e,f). PDGF-BB upregulated the expressions of cyclin B1 and cyclin D1 mRNA, and the expression levels were reduced by pioglitazone treatment (Fig. 6h). On the other hand, the increased ERK phosphorylation and decreased expression levels of p21^cip1^ and p27^Kip1^ induced by PDGF-BB remained unchanged after pioglitazone treatment (Supplementary Fig. S4g–i). These data suggest that pioglitazone suppresses VSMC proliferation via regulating the expression levels of cyclin B1 and cyclin D1.

### Pioglitazone treatment for 8 weeks improved the cardiovascular risk profile by an adiponectin-independent mechanism

**Figure 7:**
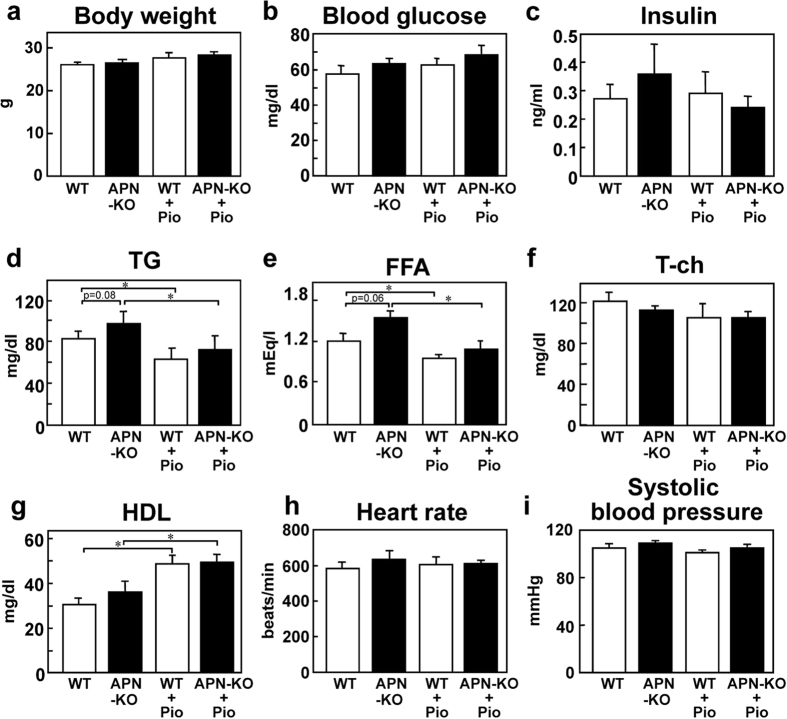
Pioglitazone treatment for 8 weeks improved the cardiovascular risk profile via an adiponectin-independent pathway (**a**) Body weight in the WT and APN-KO mice (n = 7–8). (**b**) Fasting blood glucose levels in the WT and APN-KO mice (n = 7–8). (**c**) Fasting plasma insulin levels in the WT and APN-KO mice (n = 7–8). (**d**) Fasting plasma triglyceride (TG) levels in the WT and APN-KO mice (n = 7–8). (**e**) Fasting plasma free fatty acid (FFA) levels in the WT and APN-KO mice (n = 7–8). (**f**) Fasting plasma total cholesterol (T-ch) levels in the WT and APN-KO mice (n = 7–8). (**g**) Fasting plasma high-density lipoprotein (HDL) levels in the WT and APN-KO mice (n = 7–8). (**h**) Heart rate in the WT and APN-KO mice (n = 5–6). (**i**) Systolic blood pressure in the WT and APN-KO mice (n = 5–6). Data are mean ± SEM. *P < 0.05.

Similar to the case after 3-weeks’ pioglitazone treatment, we measured the body weight, blood glucose, plasma insulin levels and the plasma lipid profile after 8 weeks of pioglitazone treatment. No significant differences were observed in the body weight, blood glucose, plasma insulin or T-ch among the four groups (Fig. 7a–c,f). The increased plasma TG and FFA levels in the APN-KO mice reduced to a degree similar to that in the WT mice after 8 weeks of pioglitazone treatment (Fig. 7d,e). In addition to the reduction in the plasma levels of these parameters, significant increase of the plasma HDL levels was noted in both the WT and APN-KO mice after 8 weeks of pioglitazone treatment (Fig. 7g). Heart rate and systolic blood pressure did not differ among the four groups after 8 weeks of pioglitazone treatment (Fig. 7h,i). These data suggest that 8 weeks of pioglitazone treatment improved the cardiovascular risk profile via an adiponectin-independent mechanism.

## Discussion

In this study, we demonstrated that pioglitazone treatment increased plasma adiponectin levels and AdipoR2 expression from the early phase of administration, resulting in the amelioration of cuff-induced neointimal formation through inhibition of VSMC proliferation. Adiponectin suppresses VSMC proliferation through both AdipoR1- and AdipoR2- AMPK pathways. In the late phase, on the other hand, the increased AdipoR2 expression seen in the early phase was blunted, and cuff-induced neointimal formation was similarly inhibited, in both the WT and APN-KO mice. Pioglitazone decreased VSMC proliferation via suppression of cyclin B1 and cyclin D1 mRNA expressions. These data suggest that the mechanism underlying the pioglitazone actions of inhibiting neointimal formation differs depending on the length of treatment. It acts via mainly an adiponectin-dependent pathway in the early phase of treatment but predominantly via an adiponectin-independent pathway in the later phase (Supplementary Fig. S5).

Increased plasma adiponectin following pioglitazone treatment for two weeks has been reported to be associated with improved insulin resistance and diabetes in ob/ob mice, but not in APN-KO/ob/ob mice^32^. Moreover, angiotensin II-induced cardiac hypertrophy and fibrosis improved following pioglitazone treatment for one week in WT mice, but not APN-KO mice^33^. Adiponectin has been reported to promptly accumulate in damaged tissues, such as wire-injured vessels^34^,^35^. These data suggest pioglitazone-induced suppression of neointimal formation mediated by adiponectin appears from the early phase of treatment. On the other hand, long-term pioglitazone treatment was associated with the inhibition of neointimal formation via an adiponectin-independent mechanisms. Although 8-weeks’ treatment with pioglitazone increased the plasma adiponectin levels to the same degree as 3-weeks’ treatment in the WT mice, cuff-induced neointimal formation improved in both the WT and APN-KO mice. The increased AdipoR2 expression observed after 3 weeks of pioglitazone treatment was blunted after 8-weeks’ treatment in both the WT and APN-KO mice. A careful comparative study of WT and VSMC-specific PPARγ-deficient mice revealed that although the plasma adiponectin levels appear to increase to the same degree in these mice, pioglitazone administration for a relatively long period inhibited the progression of atherosclerosis in the WT mice, but not in VSMC-specific PPARγ-deficient mice^16^,^17^.

Full-length adiponectin has a collagen domain, which is directly capable of binding to PDGF-BB^22^ and attenuating PDGF β-receptor autophosphorylation, resulting in inhibition of PGDF-BB-induced VSMC proliferation^22^,^36^. To investigate the effect of adiponectin on PDGF-BB-induced VSMC proliferation in the absence of direct binding with PDGF-BB and full-length adiponectin, we selected globular adiponectin to examine its effect on PDGF-BB-induced VSMC proliferation. Su *et al.* have reported that globular adiponectin inhibited PDGF-BB-induced mesangial cell proliferation without PDGF β-receptor autophosphrylation^37^. Although T-cadherin, which has been identified as one of the adiponectin receptors, specifically binds to HMW adiponectin^38^,^39^, both AdipoR1 and AdipoR2 can bind to globular adiponectin as well as full-length adiponectin^25^. Thus, we could easily observe the action of adiponectin via the AdipoRs-AMPK pathway by using globular adiponectin, instead of full-length adiponectin.

Pioglitazone activates PPARγ, which is a key transcription factor for insulin sensitivity genes. VSMC-specific PPARγ-deficient mice show accelerated atherosclerosis, which is not inhibited by pioglitazone administration^17^. Pioglitazone has been demonstrated to suppress cyclin B1 and D1 mRNA expressions via PPARγ activation *in vitro*, resulting in the inhibition of VSMC proliferation^18^. On the other hand, pioglitazone was also shown to inhibit proliferation of VSMCs through the p38 MAPK-regulated cell cycle via a PPARγ-independent pathway^40^. TZDs inhibit Ang II-induced kruppel-like factor (KLF) 5 production, resulting in the suppression of cyclin D1 expression in the VSMCs via a PPARγ-independent mechanism^41^. Thus, pioglitazone may inhibit the proliferation of VSMCs via both PPARγ-dependent and PPARγ -independent pathways.

Osman *et al.* demonstrated that treatment with 30 μM pioglitazone inhibited PDGF-BB-induced VSMC proliferation by inhibiting the mTOR activity via an AMPK-dependent mechanism^42^. Following downregulation of AMPK by target-specific siRNA, 30 μM pioglitazone inhibited ERK phosphorylation, thereby suppressing PDGF-BB-induced VSMC proliferation in an AMPK-independent manner. Moreover, both the AMPK-dependent suppression of the mTOR activity and AMPK-independent suppression of ERK phosphorylation occurred independently of PPARγ expression^42^. On the other hand, we demonstrated that 1 μM pioglitazone suppressed PDGF-BB-induced VSMC proliferation, although it had no effect on ERK phosphorylation (Fig. 6g and Supplementary Fig. S4g). It has been shown in a previous study that pioglitazone treatment at 1 μM suppresses VSMC proliferation via PPARγ activation^18^. These data suggest that the mechanisms of action of pioglitazone may differ depending on the dose; while at low concentrations, pioglitazone exerts its beneficial actions in a PPARγ-dependent manner, at higher doses, it exerts its beneficial actions via PPARγ-independent mechanisms.

Although no significant differences in the plasma HDL levels among the four study groups were observed after 3 weeks of pioglitazone treatment (Fig. 4g), 8 weeks of pioglitazone treatment was associated with increased plasma HDL levels in both the WT and APN-KO mice (Fig. 7g). Consistent with these observations, a previous study showed that pioglitazone treatment for 4 weeks had no effect on the serum HDL levels in Wistar-Kyoto rats^43^. On the other hand, Xing *et al.* demonstrated that pioglitazone treatment for 8 weeks increased the plasma HDL levels in Sprague-Dawley rats^44^. Moreover, in a randomized clinical trial, pioglitazone treatment for 24 weeks was found to be associated with a decrease of the carotid intima-media thickness in type 2 diabetics, along with increase of the plasma HDL levels^45^. Apolipoprotein (apo) A-I is produced in the liver and small intestine, and is one of the major constituents of the HDL-cholesterol fraction^46^. The promoter region of apoA-I contains a peroxisome proliferator–responsive element (PPARE), and pioglitazone has been reported to increase apoA-I production by directly enhancing PPRE-dependent transcription^47^. These data suggest that pioglitazone treatment, at least over the long term, increases the plasma HDL levels in both animal models and humans.

We could not demonstrate in this study whether long-term pioglitazone treatment exerted a stronger effect against neointimal formation than short-term pioglitazone treatment, because cuff-induced neointimal formation in WT mice was substantially inhibited by even short-term pioglitazone treatment. However, our results revealed that two pathways underlie the pioglitazone actions of inhibiting neointimal formation; an adiponectin-dependent pathway and an adiponectin-independent pathway. Pioglitazone may exert potent efficacy against neointimal formation through different mechanisms depending on the time course.

## Materials and Methods

### Animals

All animal experiment were conducted in accordance with the principles of the “Guide to the Care and Use of Experimental Animals” and were approved by the Animal Care Committee of the University of Tokyo. The APN-KO mice were generated on the original C57BL6/129sv hybrid background^20^,^32^. We backcrossed APN-KO mice with C57BL6 more than seven times. All mice were maintained under a 12/12 hour light/dark cycle. Pioglitazone was administered to the mice by admixing it with food at a concentration of 0.02% (w/w) for 3 weeks starting at 17 weeks of age, or for 8 weeks starting at 12 weeks of age (Supplemental Fig. S6). The drug was kindly provided by Takeda Chemical Institute Co., Ltd (Kanagawa, Japan).

### Cuff placement, tissue harvesting and morphometry

The mice were anesthetized by an intraperitoneal (IP) injection of 40–50 mg/kg of sodium pentobarbital. Then, an incision, about 7–10 mm in length, was made in the skin of the inguinal region under stereomicroscopic observation. The subcutaneous connective tissue was carefully removed to expose the femoral artery, followed by isolation of the femoral artery from the femoral vein by blunt dissection under stereomicroscopic observation. A polyethylene tube (inner diameter = 0.58 mm, length = 2 mm) was placed around the femoral artery. Finally, the surrounding tissue was straightened and the skin sutured. Pioglitazone administration was continued for 2 weeks after the cuff placement. Femoral arteries were then fixed *in situ* with 10% formalin and embedded in paraffin. Continuous cross-sections (5 μm) were then cut from one edge to the other of the cuffed portion. Morphometric analyses were performed on tissues stained for elastic fibers and with hematoxylin and eosin^27^. The areas of the lumen, intima and media were measured in 10 cross sections using the image analyzer software, WinROOF (Mitani Corp), and then the volume ratio of the intima to media, intimal thickness, medial thickness and luminal diameter were calculated. The examiners of the sections were blinded to the genotype of the mice. Immunohistochemical staining was performed using anti-mouse α-smooth muscle actin (Abcam). Biotinylated secondary antibody and the ABC/DAB system (Vector) were used. Two weeks after cuff placement, the mice were anesthetized and saline was infused at a constant pressure of 100 mm Hg into the left ventricle. The cuff-injured femoral artery was immersed Trizol Reagent (Invitrogen) to extract RNA.

### BrdUrd Staining

Following cuff placement, 100 μg/g of BrdUrd was administered intraperitoneally every 24 h until harvesting the femoral arteries. On the 14th day after vascular injury, the femoral arteries were perfusion-fixed in 10% formalin, harvested, and embedded in paraffin. After deparaffinization, parallel sections were immunostained with BrdUrd using a BrdUrd staining kit (Oncogene Research Products, Boston, MA). BrdUrd-labeled and -unlabeled SMCs in the neointima were counted in each section. The proliferation index was calculated by dividing the number of BrdUrd-labeled cells by the number of unlabeled cells.

### Blood sample assay

Mice were denied access to food for 16 h before the measurements. 30-μl blood samples were collected from the tail vein in accordance with the guidelines for animal experiments of the University of Tokyo. Fasting blood glucose was measured with an automatic glucometer (Glutest Ace. Sanwa Chemical Co., Japan). Plasma levels of insulin (Morinaga Co., Ltd., Japan), triglyceride, total cholesterol, free fatty acids and high-density lipoprotein (Wako Pure Chemical Industries, Ltd., Japan) were assayed by enzymatic methods. Plasma total adiponectin levels (Otsuka Pharmaceutical Co., Ltd., Tokyo, Japan) and plasma HMW adiponectin levels (ALPCO Diagnostics., USA) were measured with ELISA kits.

### Measurement of blood pressure and heart rate

Systolic blood pressure and pulse rate were measured with an automatic sphygmomanometer by the tail-cuff method in unanesthetized animals.

### Western Blot Analysis

Cell and tissue samples were homogenized in ice-cold lysis buffer (25 mM Tris-HCl (pH 7.4), 10 mM sodium orthovanadate, 10 mM EDTA, 10 mM EGTA, 1% NP-40, 1 mM phenylmethylsulfonyl fluoride, and protease inhibitor cocktail). Identical amounts of protein were resolved by 10% SDS-PAGE and transferred to a Hybond-P PVDF transfer membrane (Amersham Biosciences). Bound antibodies were detected with HRP-conjugated secondary antibodies using ECL detection reagents (Amersham Biosciences). Anti-phospho-AMPK (Thr172), anti-pan-αAMPK (α1and α2), anti-phosph-p42/44ERK, anti-ERK, and p21^cip1^ and p27^kip1^ antibodies were purchased from Cell Signaling.

### Quantitative PCR

Total RNA was isolated from the cells and tissues with Trizol (Invitrogen), or an RNeasy Mini Kit (Qiagen Co., Germany), in accordance with the manufacturer’s instructions. Less than one microgram of RNA was used for generating cDNA using random hexamers with QPCR master mix reagents (ABI). TaqMan quantitative PCR was then performed with the Applied Biosystems 7900HT Fast Realtime PCR system (Applied Biosystems) using TaqMan Gene Expression Assays or SYBR Green. TaqMan Gene Expression Assays used were as follows; AdipoR1, AdipoR2, CDKN1b. The primers used were as follows; Cyclin B1: 5′-CTGGGTCGGGAAGTCACTGGAAAC-3, 5′-GCAGCATCTTCTTGGGCACACA-3′; Cyclin D1: 5′-AGGCGGAGGAGAACAAACAGATCA-3′, 5′-AGAGGAAGCGTGTGAGGCGGTAGTA-3; GAPDH: 5′- AATGAAGGGGTCATTGATGG-3′,5′-AAGGTGAAGGTCGGAGTCAA-3′. The expression levels of each of the transcripts were normalized to the constitutive expression levels of β-actin and GAPDH mRNA.

### siRNA transfection

Human aortic SMCs (HASMCs) and the smooth muscle cell growth medium (SmGM2) were purchased from Takara. For all the experiments, SMCs at passages 4–7 were used. HASMCs were seeded at 3 × 10^3^ cells on to a 96-well dish; 24 hours after the plating, siControl (1.5 pmol; Santa Cruz), siAdipoR1 (1.5 pmol; Santa Cruz), siAdipoR2 (1.5 pmol; Santa Cruz), or siAMPK (α1and α2) (1.5 pmol; Santa Cruz) diluted with Opti-MEM^®^ was transfected into the cells with Lipofectamine RNAiMAX (Invitrogen). The cells were used for the assay 24 hours after the transfection.

### Cell proliferation assay

For the cell counting assay, HASMCs were seeded at 3 × 10^3^ cells on to a 96-well dish; 24 hours after the plating, the cells were serum-starved to render them quiescent by replacing the medium with DMEM containing 0.2% FBS. The quiescent SMCs were then preincubated with globular adiponectin (1 μg/mL or 3 μg/mL) or pioglitazone (1 μM or 10 μM) for 30 min. PDGF-BB (10 ng/mL) was added to stimulate the cells for 24 hours. These cells were incubated with 10 μl of CCK (cell counting kit)-8 (DOJINDO) or WST-1 (Roche) solution for 2 hours before conducting the measurements. We measured the absorbance at 450 nm using a microplate reader. 10 μl of BrdU (Roche) solution was added these cells simultaneously with PDGF-BB. We measured BrdU incorporation by chemiluminescent assay.

### Endothelium-denuded aorta

Mice were sacrificed by an overdose of sodium pentobarbital. The thoracic aorta was gently excised, put in 0.2%FBS/DMEM, and the fat tissue around the aortic region was removed. The aorta was opened longitudinally with scissors, and the intimal surface of the vessels was delicately scrubbed with a cotton swab to remove the endothelium. The denuded aorta was stimulated with globular adiponectin 2 hours.

### Statistical analysis

Values are expressed as means ± S.E. Statistical analyses were performed by analysis of variance (ANOVA), and post hoc analysis was performed by the Turkey-Kramer method. P values of <0.05 were considered to indicate a statistically significant difference.

## Additional Information

**How to cite this article**: Kubota, T. *et al.* Pioglitazone Ameliorates Smooth Muscle Cell Proliferation in Cuff-Induced Neointimal Formation by Both Adiponectin-Dependent and -Independent Pathways. *Sci. Rep.* **6**, 34707; doi: 10.1038/srep34707 (2016).

## Supplementary Material

Supplementary Information
