# Supplementary material for: Pioglitazone Ameliorates Smooth Muscle Cell Proliferation in Cuff-Induced Neointimal Formation by Both Adiponectin-Dependent and -Independent Pathways
Source: Sci Rep. 2016 Oct 5;6:34707. doi: 10.1038/srep34707 (PMC5050439; doi:10.1038/srep34707)
Supplement: Supplementary Information [file srep34707-s1.pdf]

SUPPLEMENTAL INFORMATION

Pioglitazone Ameliorates Smooth Muscle Cell Proliferation in Cuff-Induced Neointimal Formation by Both Adiponectin-Dependent and -Independent Pathways

Tetsuya Kubota,[1,2,3,4#] Naoto Kubota,[1,2,3,5#,*] Hiroyuki Sato,[1#] Mariko Inoue,[1,3] Hiroki Kumagai,[1] Tomokatsu Iwamura,[1] Iseki Takamoto,[1] Tsuneo Kobayashi,[6] Masao Moroi,[4] Yasuo Terauchi,[7] Kazuyuki Tobe,[8] Kohjiro Ueki,[1] Takashi Kadowaki[1*]

[1] Department of Diabetes and Metabolic Diseases, Graduate School of Medicine, University of Tokyo, Tokyo 113-8655, Japan

[2] Laboratory for Metabolic Homeostasis, RIKEN Center for Integrative Medical Sciences, Kanagawa, 230-0045, Japan

[3] Department of Clinical Nutrition, National Institute of Health and Nutrition, Tokyo 162-8636, Japan

[4] Division of Cardiovascular Medicine, Toho University Ohashi Medical Center, Tokyo 153-8515, Japan

[5] Department of Clinical Nutrition Therapy, University of Tokyo, Tokyo 113-8655, Japan

[6] Department of Physiology and Morphology, Institute of Medicinal Chemistry, Hoshi University, Tokyo 142-8501, Japan

[7] Department of Diabetes and Endocrinology, Yokohama City University, School of Medicine, Kanagawa 236-0004, Japan

[8] First Department of Internal Medicine, Faculty of Medicine, University of Toyama, Toyama, 930-0194, Japan

[#] All of the authors contributed equally to this work.

*To whom correspondence should be addressed: Naoto Kubota, M.D., Ph.D., and Takashi Kadowaki, M.D., Ph.D., Department of Diabetes and Metabolic Diseases, Graduate School of Medicine, University of Tokyo, 7-3-1 Hongo, Bunkyo-ku, Tokyo 113-8655, Japan.

Figure S1

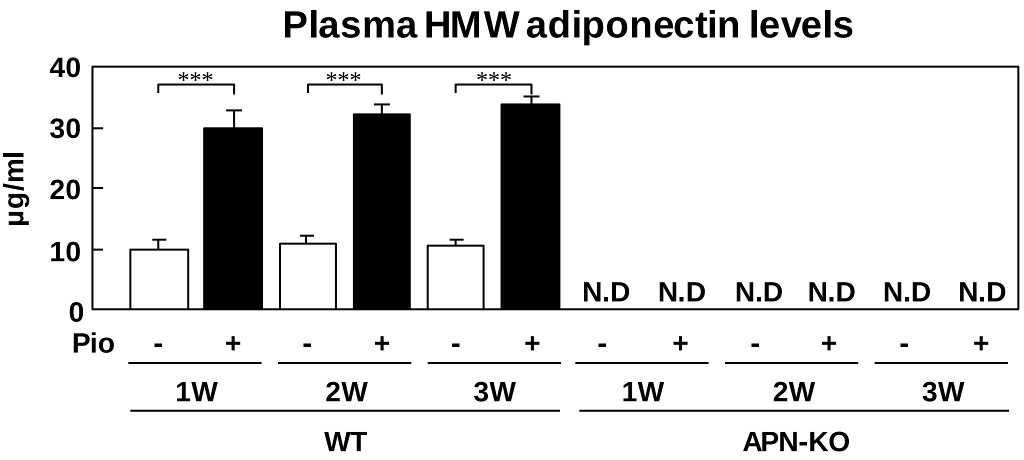

Figure S1. Increased plasma HMW adiponectin levels began to be observed from 1 week onward after the start of pioglitazone treatment in the WT mice, but not in the APN-KO mice. Plasma HMW adiponectin levels after pioglitazone treatment in the WT and APN-KO mice (n = 4-5). N.D. is “not-detected”. Data are mean ± SEM. ***, $P < 0.001$.

Figure S2

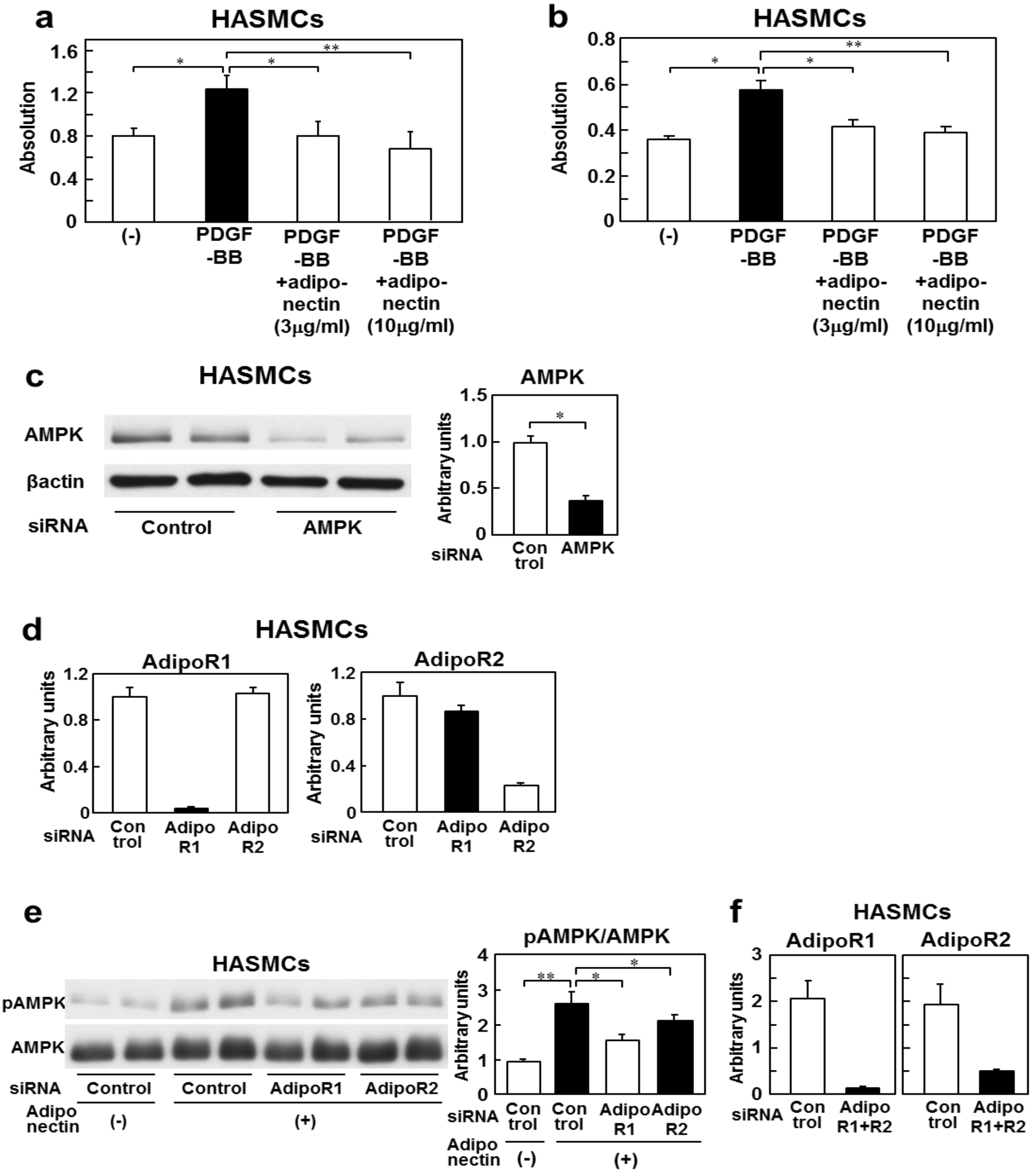

Figure S2. The expression levels of AMPK protein, AdipoR1 and AdipoR2 mRNA were reduced by specific siRNA transfections into the HASMCs. Effect of globular adiponectin on PDGF-BB-induced cell viability in the HASMCs using the CCK-8 assay **(a)** and WST-1 cell proliferation assay (**b**) (n = 4-8) (**c**) siAMPK downregulated AMPK protein expression (n = 4). (**d**) siAdipoR1 and siAdipoR2 downregulated AdipoR1 and AdipoR2 mRNA expression, respectively (n = 4). (**e**) Downregulation of AdipoR1 or AdipoR2 partially reduced AMPK phosphorylation (n = 4). (**f**) siAdipoR1 plus siAdipoR2 downregulated the expressions of both AdipoR1 and AdipoR2 mRNA (n = 4). Data are mean ± SEM. *, $P < 0.05$; **, $P < 0.01$; ***, $P < 0.001$.

Figure S3

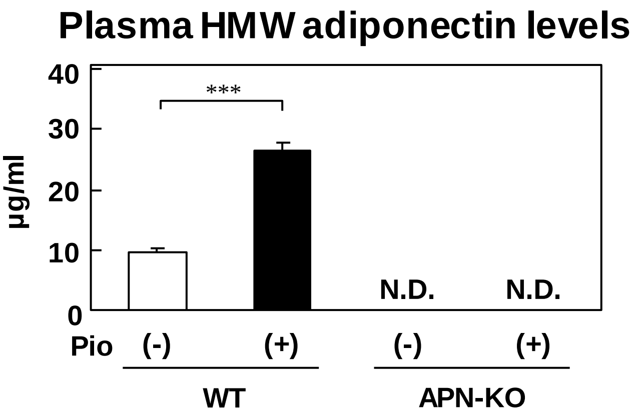

Figure S3. Increased plasma HMW adiponectin levels were observed after 8 weeks of pioglitazone administration in the WT mice, but not in the APN-KO mice. Plasma HMW adiponectin levels after 8 weeks of pioglitazone treatment in the WT and APN-KO mice (n = 5). N.D. is "not-detected". Data are mean ± SEM. ***, $P < 0.001$.

Figure S4

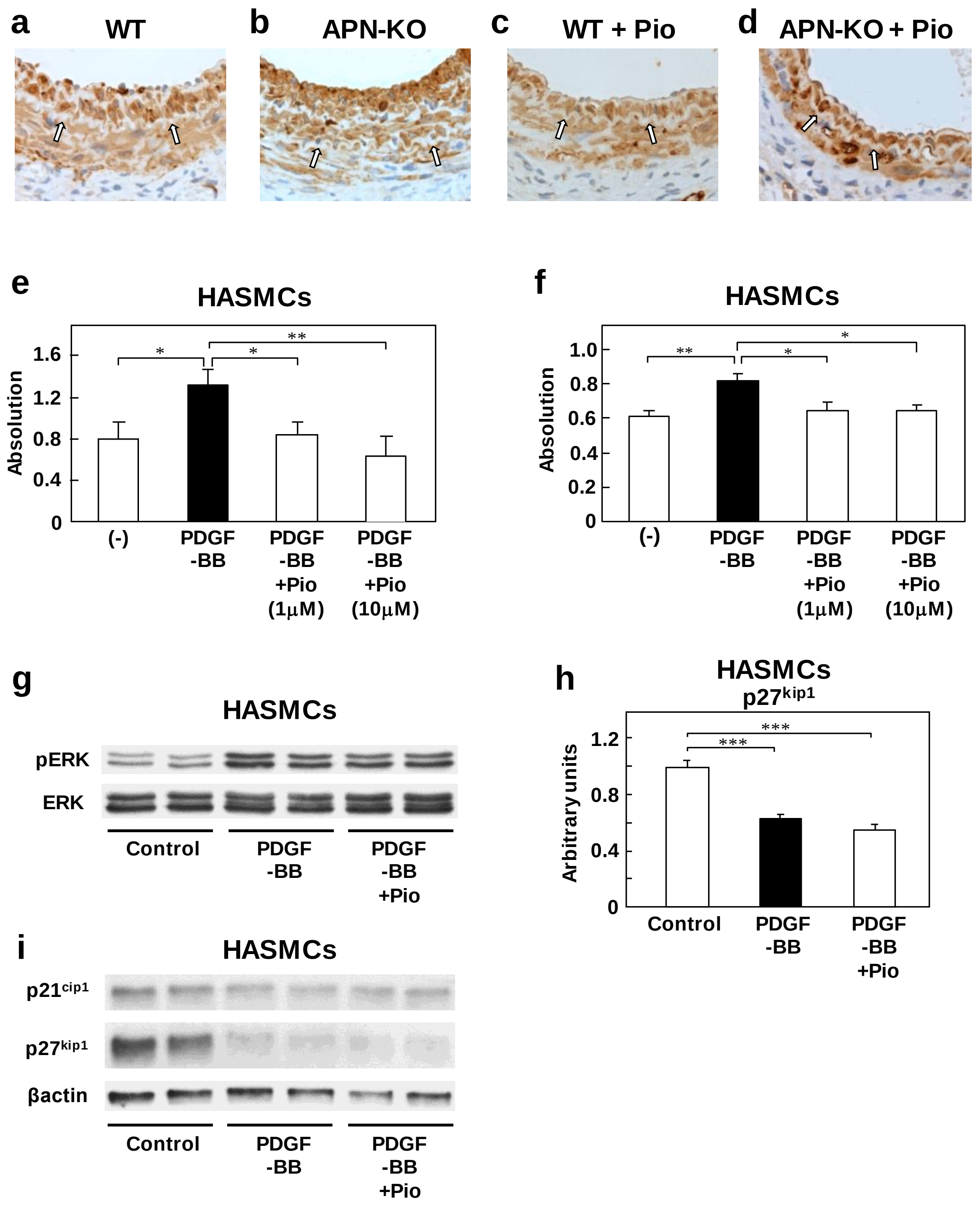

Figure S4. Neointima formed in response to cuff-induced injury was composed predominantly of VSMCs after 8 weeks of treatment with pioglitazone. Representative immunohistochemical staining for alpha smooth muscle actin of injured vessels obtained from WT (**a**), APN-KO (**b**), WT+Pio (**c**) and APN-KO+Pio (**d**) mice after 8-weeks' treatment with pioglitazone. Effect of pioglitazone on PDGF-BB-induced cell viability in the HASMCs using the CCK-8 assay **(e)** and WST-1 cell proliferation assay **(f)** (n = 4-6). (**g**) Phosphorylation of ERK was induced by PDGF-BB, however, this effect was not reversed by pioglitazone (n = 4). (**h**) PDGF-BB decreased the expression of p27^{kip1} mRNA, however, the expression was not restored by pioglitazone (n = 4). (**i**) PDGF-BB decreased the expression levels of both p21^{cip1} and p27^{kip1} protein, however, the expressions were not restored by pioglitazone (n = 4). Arrows show the internal elastic lamina. Data are mean ± SEM. ***, $P < 0.001$.

Figure S5

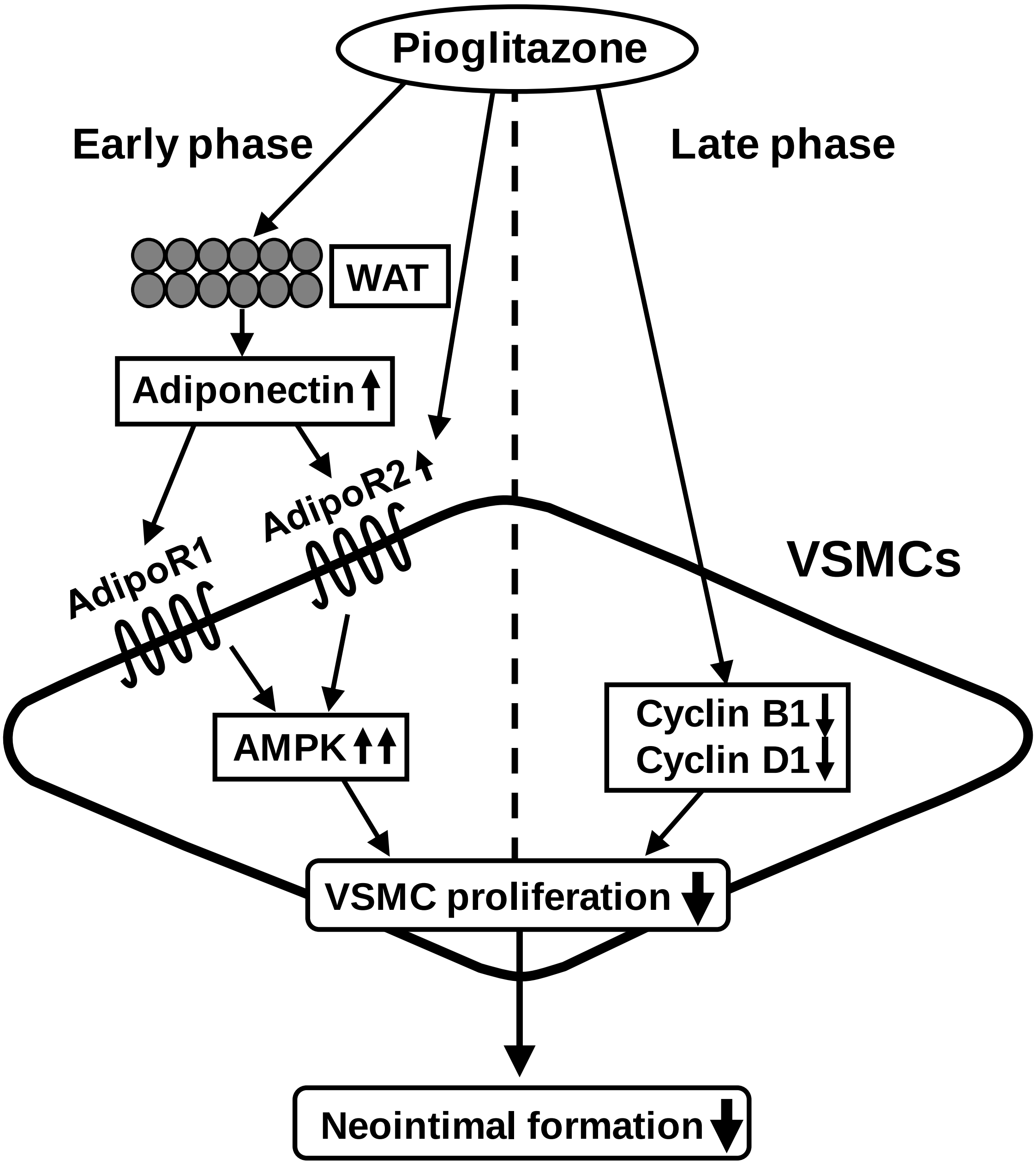

Figure S5. Schema of pioglitazone-induced suppression of neointimal formation by both adiponectin-dependent and adiponectin-independent mechanisms. Pioglitazone increased the plasma adiponectin levels from the early phase of treatment and inhibited the proliferation of VSMCs via an adiponectin-dependent pathway, thereby inhibiting neointimal formation. Adiponectin suppressed the proliferation of VSMCs through AdipoR1- and AdipoR2- mediated AMPK activation. On the other hand, long-term treatment with pioglitazone suppressed VSMC proliferation via an adiponectin-independent pathway by reducing the expressions of cyclin B1 and D1, thereby inhibiting neointimal formation.

Figure S6

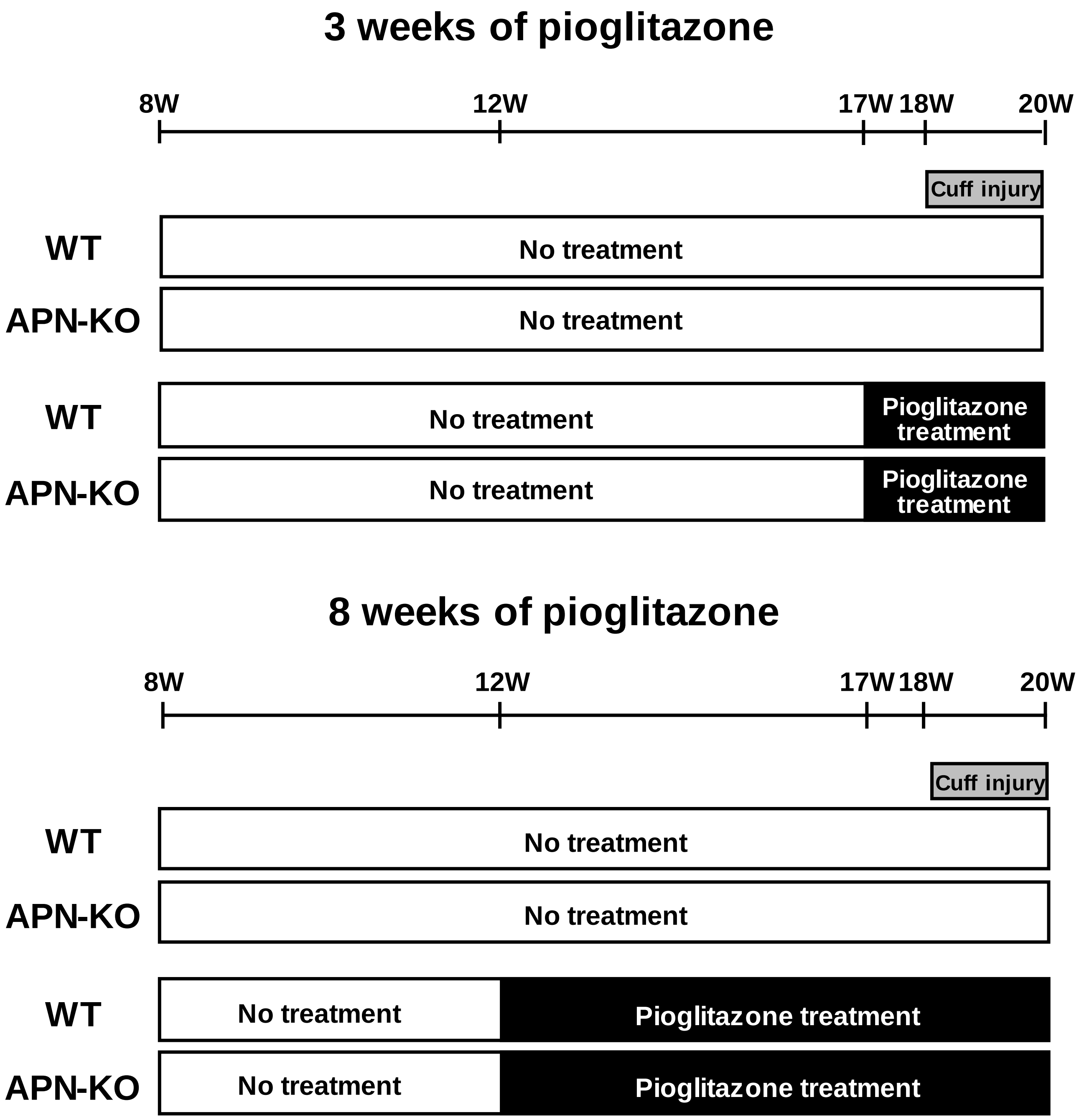

Figure S6. Experimental protocol of pioglitazone treatment for 3 or 8 weeks in the WT and APN-KO mice. Pioglitazone was administered to the mice for 3 weeks starting at 17 weeks of age, or for 8 weeks starting at 12 weeks of age.
